# Hypoglycemic Activity through a Novel Combination of Fruiting Body and Mycelia of *Cordyceps militaris* in High-Fat Diet-Induced Type 2 Diabetes Mellitus Mice

**DOI:** 10.1155/2015/723190

**Published:** 2015-07-16

**Authors:** Sung-Hsun Yu, Szu-Yu Tina Chen, Wei-Shan Li, Navneet Kumar Dubey, Wei-Hong Chen, Jiunn-Jye Chuu, Sy-Jye Leu, Win-Ping Deng

**Affiliations:** ^1^Graduate Institute of Medical Sciences, School of Medicine, Taipei Medical University, Taipei 110, Taiwan; ^2^Stem Cell Research Center, Taipei Medical University, Taipei 110, Taiwan; ^3^Graduate Institute of Biomedical Materials and Tissue Engineering, College of Oral Medicine, Taipei Medical University, Taipei 110, Taiwan; ^4^Institute of Biotechnology, College of Engineering, Southern Taiwan University of Science and Technology, Yongkang District, Tainan, Taiwan; ^5^Department of Microbiology and Immunology, School of Medicine, Taipei Medical University, Taipei 110, Taiwan

## Abstract

Diabetes mellitus (DM) is currently ranked among leading causes of death worldwide in which type 2 DM is reaching an epidemic proportion. Hypoglycemic medications for type 2 DM have either proven inadequate or posed adverse effects; therefore, the Chinese herbal products are under investigation as an alternative treatment. In this study, a novel combination of fruiting body and mycelia powder of herbal *Cordyceps militaris* number 1 (CmNo1) was administered to evaluate their potential hypoglycemic effects in high-fat diet- (HFD-) induced type 2 DM in C57BL/6J mice. Body weight, fasting blood glucose (FBG), oral glucose tolerance test (OGTT), and blood biochemistry indexes were measured. Results indicated that CmNo1 lowered the blood glucose level by increasing insulin sensitivity, while no change in body weight was observed. Increased protein expression of IRS-1, pIRS-1, AKT, pAKT, and GLUT-4 in skeletal muscle and adipose tissue was found indicating restoration of insulin signaling. Additionally, PPAR-*γ* expression in adipose tissue restored the triglyceride and cholesterol levels. Finally, our results suggest that CmNo1 possesses strong hypoglycemic, anticholesterolemic, and antihypertriglyceridemic actions and is more economical alternate for DM treatment.

## 1. Introduction

Diabetes mellitus (DM) is one of the most common chronic diseases worldwide. Type 2 DM, accounting for 95% of all cases, is a complex metabolic syndrome characterized by hyperglycemia resulting from either insulin resistance or inadequate insulin secretion. Being a metabolic disorder, type 2 DM is associated with comorbidities, encompassing obesity, heart disease, and stroke [1]. Lifestyle and dietary factors, along with genetic predisposition, are integral to the development of both diabetes and accompanying complications. The growing number of diagnoses of type 2 DM is correlated with increased consumption of low-cost, high-fat, and high-calorie diets [2]. Particularly, obesity is one of the leading causative factors of type 2 DM in both human and animal models.

Type 2 DM can be examined in rodent model created by diabetogenic agents, for example, streptozotocin (STZ) and nicotinamide [3, 4], or in inbred diabetic rodent models with inherited hyperglycemia, such as db/db and ob/ob mice [5–7]. In addition, high-fat diet (HFD) fed mice are another promising model to develop the manifestations of type 2 DM with diminished insulin function, insulin resistance, and hence the overproduction of glucose in mice [8]. The advantage of HFD is that it imitates the natural history and metabolic characteristics of the human syndrome while remaining responsive to pharmaceutical treatment [9]. Mice fed with HFD (60% fat kcal) have been found to promote insulin resistance [10–12], a phenomenon in which insulin mediation is malfunctioned, but the physiological mechanisms of insulin production are normal [13]. It describes impaired insulin utilization in tissues albeit the sufficient amount of it is present in the body. Therefore, there is an imbalance between hepatic glucose production and its utilization by tissue [14, 15]. Currently, several drugs are recommended for treatment of type 2 DM; however, many of them are associated with side effects like gastrointestinal and cardiovascular events [16, 17]. For instance, thiazolidinediones consumption also leads to higher risks of health failure, fracture, heart attack, and bladder cancer [18]. Treatment with *α*-glucosidase inhibitors leads to higher risk of nausea, vomiting, and diarrhea [19, 20]. Moreover, the intake of these drugs results in both weight gain and increased risk of low blood sugar. Therefore, a natural and inexpensive medication with no adverse effects after long-term consumption in diabetic patients is needed.

In traditional Chinese medicine, the* Cordyceps*, or “winter worm and summer grass,” a parasitic fungus, has been extensively utilized. Previous studies demonstrated that it can alter immune response [21] and inhibit tumor growth [21–23]. In addition,* Cordyceps *species has been shown to exhibit hypoglycemic activity [24–26]. Its ability to lower blood glucose has been attributed to the components comprising the high polysaccharide content extracted from mycelia [27]. Previous studies also indicate that* Cordycep*s can protect cells through mechanisms involving hepatic glucokinase, hexokinase, and glucose-6-phosphate dehydrogenase [26–28]. Among the* Cordyceps* species, the hypoglycemic action of fruiting body, and mycelium, each of them separately has already been analyzed and identified. Although many strains of* Cordyceps* possess remarkable therapeutic activities, the* C. militaris* (*CM*) is currently regarded as an alternative of well-known* C. sinensis *in being widely available, cheaper, and strongly antihyperglycemic. Extracts from the fruiting body of* CM* have demonstrated greatest hypoglycemic effect compared to other strains and component, but owing to host specificity and rarity the fruiting body of* CM* is very expensive [29, 30]. In another study, only mycelium-mediated hypoglycemic effect of* CM* in diet-STZ-induced DM in rats has been reported [31].

Based on the above earlier reports, it may be advocated to use only mycelia instead of fruiting body. However, keeping in the view of facts about hypoglycemic efficacy and its economical perspective, we represented the first report on hypoglycemic effect by combining fruiting body and mycelia of* CM* in HFD-induced type 2 DM mice. We have also examined the anticholesterolemic and antihypertriglyceridemic profile.

## 2. Materials and Methods

### 2.1. Preparation of* Cordyceps militaris* Number 1 (CmNo1) Crude Powder

The commercially pulverized crude powder of the combined fruiting body and mycelium of* CM*, denoted by CmNo1, used in this study was provided by Mu Cho BioTechnology Co., Ltd. (Taipei, Taiwan). The CmNo1 was administered to mice at a dose of 360 mg/kg/day.

### 2.2. Animal Preparation

C57BL/6J mice were purchased from National Laboratory Animal Center, Taipei, Taiwan, and were maintained at Laboratory Animal Center, Taipei Medical University (TMU). All the animal care and use protocols were in accordance with guidelines of TMU Institutional Animal Care and Use Committee (IACUC). The 3 groups, each of 6-week-old male C57BL/6J mice, that is, control, type 2 diabetic (DM), and CmNo1-treated DM (CmNo1-DM) mice, were housed separately and maintained on a 12 hr light/dark cycle at temperature of 24°C. Control mice were fed with normal chow (LabDiet 5010, 5.5% fat), while DM and CmNo1-DM group were fed with high-fat diet (HFD, 58Y1, DIO Rodent Purified Diet, TestDiet) with 61.6% fat (3.140 Kcal).

### 2.3. Examination of Fasting Blood Glucose (FBG)

Blood samples were collected from tail-vein of control, 6 months HFD administered DM, and CmNo1-treated-DM (CmNo1-DM) mice and FBG was measured by glucose oxidase strips (Easytouch, Taiwan).

### 2.4. Oral Glucose Tolerance Test (OGTT)

The OGTT was performed on weekly basis in the control, DM, and CmNo1-DM mice during 8 consecutive weeks of CmNo1 treatment using a standard method [32]. Mice were fasted overnight followed by oral administration of 3 g/kg D-glucose. Blood samples were collected from each group at 0, 30, 60, 90, 120, and 180 minutes relative to the start of the oral glucose administration for measuring blood glucose levels. The FBG was measured by glucose oxidase strips (Easytouch, Taiwan). The area under the glucose tolerance curve (ΔAUC_glucose_) was calculated using the trapezoidal rule to determine the integrated glucose response to the glucose challenge.

### 2.5. Determination of Body Weight

C57BL/6J mice were first fed HFD to induce type 2 DM. After 6 months, CmNo1-DM group mice on HFD were orally administered CmNo1 (360 mg/kg) and a combination of fruiting body and mycelium, for eight consecutive weeks, while DM group mice on HFD were administered vehicle (double-distilled water) and control group were fed normal chow. Thereafter, the body weight of control, DM, and CmNo1-DM mice was recorded weekly for eight weeks with an electrobalance (Excell, BH-1200).

### 2.6. Measurement of Biochemistry Indexes

The blood samples were obtained from the retroorbital sinus of mice (4 times): twice before and after CmNo1 treatment. Samples were centrifuged and 25 *μ*L of serum was collected from each of them. Biochemistry indexes and insulin levels were measured using the biochemistry analyzers and insulin ELISA kit (MercodiaAB, Sweden). Serum levels of cholesterol and triglyceride were measured by chemistry analyzer (Fuji Dri-Chem 4000i).

### 2.7. Western Blot Analysis

After eight-week CmNo1 treatment, C57BL/6J mice from each group were sacrificed. Skeletal muscles, specifically thigh and gastrocnemius (calf), and intraperitoneal adipose tissue were surgically extracted and grinded. For protein extraction, tissues were suspended in 100 *μ*L 1x RIPA lysis buffer (catalogue number 20–188, Millipore, USA), protease inhibitor cocktail set III, EDTA-free (catalogue number 539134, Millipore, USA), and phosphatase inhibitor (Na_3_VO_4_) and were sonicated. After 20 minutes of incubation on ice, samples were centrifuged at 12000 rpm for 40 minutes at 4°C and supernatant was collected for quantification. The protein samples were resolved on 10% SDS-PAGE gel and transferred to a PVDF (Polyvinylidene Fluoride, Amersham Hybond-P, GE Healthcare, UK) membrane. After blocking, membranes were incubated for 1 h at room temperature in PBST buffer with the anti-glucose transporter type 4 (GLUT-4) (1 : 1000; GeneTex, Irvine, CA, USA), anti-insulin receptor substrate-1 (IRS-1; 1 : 1000, GeneTex, Irvine, CA, USA), anti-pIRS-1 (1 : 1000; Millipore, Germany), anti-protein kinase B (AKT) (1 : 5000; GeneTex, Irvine, CA, USA), anti-pAKT (1 : 1000; Cell Signaling Technology, USA), and anti-peroxisome proliferator-activated receptor *γ* (anti-PPAR-*γ*; 1 : 1000, Cell Signaling Technology, USA) antibodies. This was followed by 4 times of wash for 10 minutes each at room temperature. Horseradish peroxidase-conjugated anti-rabbit and anti-mouse IgG secondary antibody (1 : 10000; Jackson ImmunoResearch, West Grove, PA, USA) was diluted in (0.01 M  K_2_HPO_4_, 0.15 M NaCl, 0.05% Tween-20, and pH 7.0) and incubated with blots for 1 h at room temperature. Immunoreactivity expressions of GLUT-4, IRS-1, pIRS-1, AKT, pAKT, and PPAR-*γ* were measured by developing blots using ECL plus-kit (Amersham Pharmacia, USA). Blots were visualized by UVP BioSpectrum imaging system while their densities were analyzed with VisionWorks LS software.

### 2.8. Statistical Analysis

All the values were represented as standard error of mean (± SEM) of 6 mice for each group. The differences between 3 groups (control, DM, and CmNo1-DM) were estimated by Student's *t*-test (SigmaPlot Version 10.0). Each value represents the mean ± SEM (*n* = 6). Symbols specify significant difference from DM with *∗*, *∗∗*, and *∗∗∗* indicating *p* < 0.05, *p* < 0.01, and *p* < 0.001, respectively.

## 3. Results

### 3.1. Assessment of Fasting Blood Glucose (FBG)

**Figure 1:**
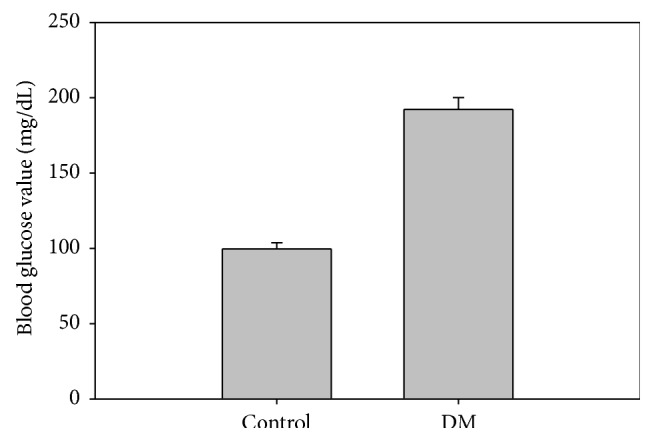
Determination of fasting blood glucose (FBG) in mice. FBG was measured in control and HFD administered DM mice for 6 months.

In order to establish a diabetes mellitus (DM) mouse model, C57BL/6J mice were fed high-fat diet (HFD) for 6 months. As a result, the FBG value in DM mice was vigorously increased to 192.33 mg/dL while control showed a normoglycemic value of 99.67 mg/dL (Figure 1).

### 3.2. Oral Glucose Tolerance Test (OGTT) after Treatment with CmNo1

**Figure 2:**
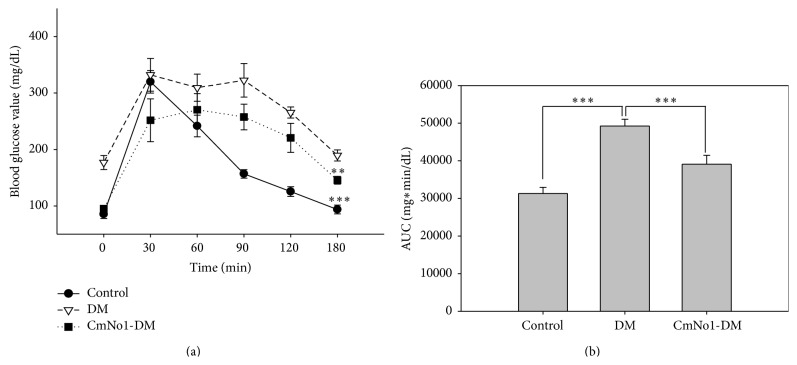
Assessment of glucose tolerance. (a) A plot of time-dependent glucose tolerance curves in control, DM, and eight-week CmNo1-treated DM mice (CmNo1-DM). OGTT followed overnight fasting, blood samples collection, and finally 3 g/kg D-glucose oral administration to determine the glucose level at 0, 30, 60, 90, 120, and 180 min. (b) Area under the curve (AUC) was calculated to determine glucose tolerance. Data is expressed as the means ± SEM (*n* = 6/group). Symbols specify significant difference from DM with *∗∗* and *∗∗∗* indicating *p* < 0.01 and *p* < 0.001, respectively.

In the rodent models, the OGTT is most widely conducted to evaluate whether the mice are glucose intolerant and diabetic. In our result, compared to control, the HFD-fed DM group had higher blood glucose level during 180 min period. The time-dependent blood glucose reduction curve demonstrated that CmNo1-DM mice displayed lower blood glucose levels than DM group (Figure 2(a)). To further determine the improvement of glucose tolerance following long-term CmNo1 treatment, the area under the curve (AUC) was calculated (Figure 2(b)). The AUC in DM mice was much increased compared to control mice; meanwhile, the increased AUC in DM mice was reduced by CmNo1 treatment.

### 3.3. No Effect of CmNo1 on Body Weight

**Figure 3:**
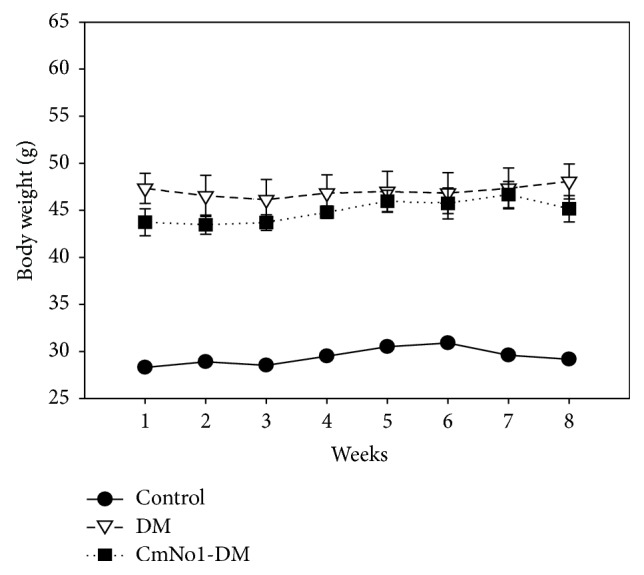
Maintenance of body weight. Body weight in control (●), type 2 diabetes mellitus (DM) (*▽*), and CmNo1-treated DM (CmNo1-DM) (■) mice was examined. Mice were first exposed to high-fat diet (HFD) for six months to induce C57BL/6J mice with type 2 DM, which were then treated with CmNo1 for eight weeks. The body weight of all three groups was recorded to note any significant change. Data is expressed as the means ± SEM (*n* = 6/group).

The HFD has been associated with a gain in body weight which is an identified risk factor for development of type 2 DM. Our results showed significantly increased body weight in DM group, compared to control group (Figure 3). However, the treatment of CmNo1 showed no effect on body weight compared to DM group.

### 3.4. Effect of CmNo1 on Serum Insulin Levels

**Figure 4:**
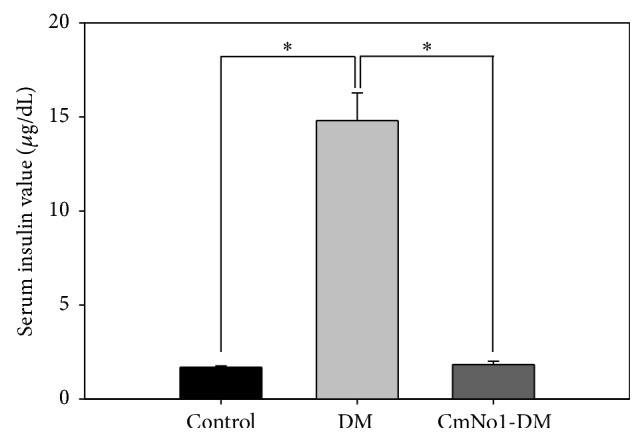
Serum insulin levels. After eight-week CmNo1 treatment, blood was drawn from overnight-fasted mice, and serum was analyzed by ELISA for insulin levels compared to control and DM mice. Data is expressed as the means ± SEM (*n* = 6/group). Symbols specify significant difference from DM with *∗* indicating *p* < 0.05.

To investigate the restoration effect of CmNo1 treatment on insulin sensitivity, serum insulin levels were measured in subjects after overnight fasting. The serum insulin values in DM mice were increased nearly 9-fold compared to those in control mice (Figure 4). In contrast, after CmNo1 treatment (CmNo1-DM mice), the increased insulin levels in DM mice were diminished to values similar to those of control mice.

### 3.5. Expression of Specific Protein Markers in Skeletal and Adipose Tissues

**Figure 5:**
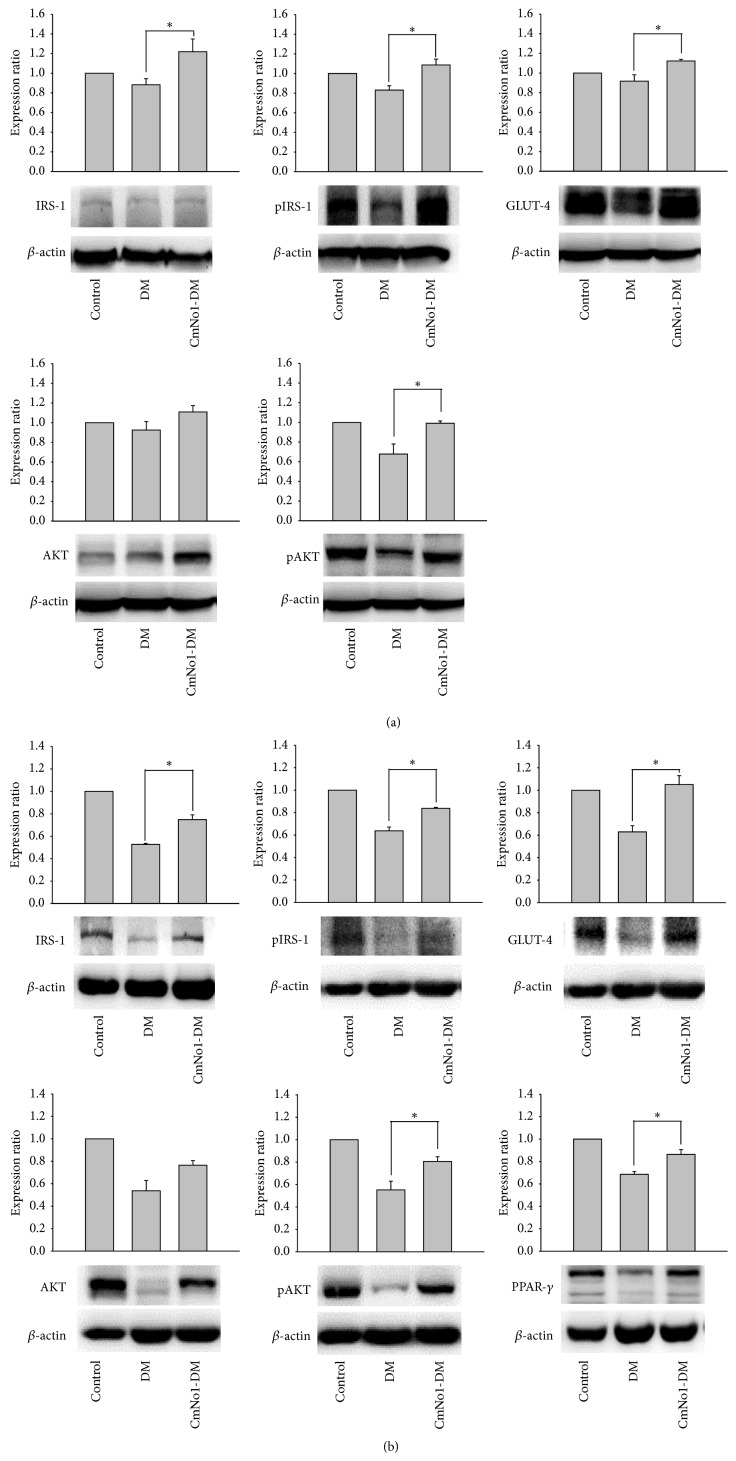
Specific protein expression of skeletal muscle and adipose tissue. (a) The protein expression of insulin receptor substrate-1 (IRS-1), phosphorylated IRS-1 (pIRS-1), protein kinase B (AKT), phosphorylated AKT (pAKT), and glucose transporter type 4 (GLUT-4) in skeletal muscle was measured. *β*-actin was used as a loading control in Western blot analysis and the measurement was expressed as a ratio of specific protein/*β*-actin expression. (b) Analysis for IRS-1, pIRS-1, AKT, pAKT, GLUT-4, and peroxisome proliferator-activated receptor *γ* (PPAR-*γ*) in adipose tissue. Data is expressed as the means ± SEM of *n* = 6 samples. Symbols specify significant difference from DM with *∗* and *∗∗* indicating *p* < 0.05 and *p* < 0.01, respectively.

Skeletal muscle [33] and adipose tissue [34] are two major consumers of glucose and an essential regulator of insulin for glucose uptake. To evaluate insulin sensitivity through the expression of specific skeletal muscles and adipose tissue proteins involved in regulating insulin signaling pathway, including insulin receptor substrate-1 (IRS-1), protein kinase B (AKT), and glucose transporter type 4 (GLUT-4), the tissues were harvested and homogenized. Western blotting was conducted with *β*-actin as a loading control. Results showed that protein expression of IRS-1, phosphorylated IRS-1 (pIRS1), phosphorylated AKT (pAKT), and GLUT-4 in skeletal muscle was significantly decreased in DM mice but increased in CmNo1-DM group after 8-week CmNo1 treatment (Figure 5(a)). Similarly, we also demonstrated upregulation in protein expression of IRS-1, pIRS-1, GLUT-4, pAKT, and adipogenic marker peroxisome proliferator-activated receptor *γ* (PPAR-*γ*) in adipose tissues from CmNo1-DM group compared to those from DM group (Figure 5(b)).

### 3.6. Effect of CmNo1 on Triglyceride and Cholesterol Levels

**Figure 6:**
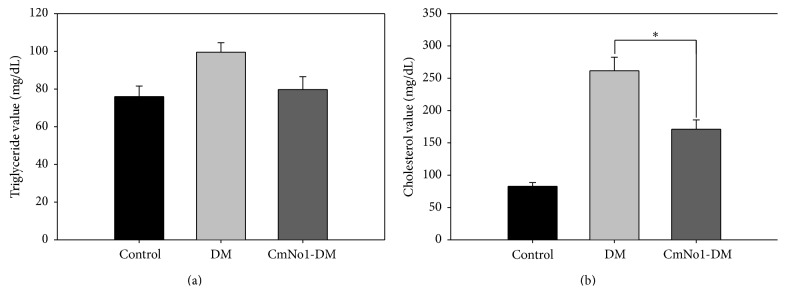
Triglyceride and cholesterol levels. (a) Triglyceride and (b) cholesterol levels were measured in blood samples drawn from control, DM, and CmNo1-treated DM mice after overnight fasting. Data is expressed as the means ± SEM (*n* = 6/group). Symbols specify significant difference compared to DM with *∗* indicating *p* < 0.05.

PPAR-*γ* plays a key role in regulating insulin sensitization as well as improving cholesterol efflux and triglyceride lowering [35]; hence the levels of triglyceride and cholesterol levels were then detected. In Figure 6, as per our prediction, results demonstrated that both triglyceride and cholesterol were significantly reduced after CmNo1 treatment.

## 4. Discussion

As per the death toll, a noncommunicable disease, diabetes mellitus (DM), has been ranked as “third killer” worldwide [31]. Several animal models have been developed and extensively employed in the diabetes research [36] out of which the high-fat diet (HFD) regimen in mice was utilized to induce type 2 DM. This approach mimics the availability of fat-rich diet in our modern society, thereby contributing to cause of diabetes in humans. For DM treatment, Chinese herbal medicines have been reported to possess antidiabetic properties [32] in which* Cordyceps* species like* C. sinensis* (*CS*) have been most studied and represented its beneficial effect on diabetic mammals. Though* CS* is well-known for the treatment of DM,* C. militaris* (*CM*) has drawn more attention in recent years due to its higher availability, lower price (10 times), and more potential pharmacologic properties [37, 38]. Moreover, the natural resources of* CS* are declining, so the artificial cultivation of* CM* would be a better alternative. A few studies have demonstrated the effect of each of fruiting body and mycelia of* CM* strain on glucose metabolism [31, 38] in which their polysaccharide fraction has been attributed to hypoglycemic activity. In a seminal study, it has been reported that, compared to fruiting bodies of same origin, the mycelia contain not only the higher amount of polysaccharides but also adenosine [39]. In another study, higher carbohydrate content has been demonstrated in mycelia than fruiting bodies [40], while Zhang's study demonstrated higher potency of* CM* fruiting body than* CM* mycelia [38]. However, the comparative quantification of higher polysaccharide and other components either in fruiting body or in mycelia remains questionable. Another critical issue is that the fruiting bodies of* CM* are very expensive due to host specificity and rarity in nature [41]. In this study, the fruiting body and mycelia of* CM* were first combined (designated as CmNo1) and* in vivo* efficacies along with detailed therapeutic mechanisms of CmNo1 towards antihyperglycemic, anticholesterolemic, and antihypertriglyceridemic activities were examined.

CmNo1 was grown on specialized organic rice media in a sterile environment resulting in high production of polysaccharide (209.81 mg/g). The other components such as cordycepin and adenosine were also determined at high levels as 8.23 mg/g and 0.68 mg/g, respectively. Cordycepin (3′-deoxyadenosine) is a biometabolite component of* CM* which possess antibacterial [42], antimetastatic [43], immunomodulating [44], antifungal [45], and insecticidal [46] activity along with suppressing the type 2 DM regulating genes [47]. Besides, adenosine in* Cordyceps* species is associated with regulation of coronary blood flow [48] and immunomodulation [47]. Compared to Dong's study [31], we have demonstrated higher polysaccharide content, suggesting enhanced hypoglycemic efficacy of* CM.* The novel finding of this study is the combinational effect of fruiting body and mycelia of* CM* in terms of substantial antidiabetic, anticholesterolemic, and antihypertriglyceridemic characteristics along with ensuring economic viability.

It is also indicated that CmNo1 strongly improved type 2 DM through lowering blood glucose (Figure 2) without any change in body weight (Figure 3) during treatment. Currently recommended second-tier drugs for DM treatment such as basal insulin, thiazolidinedione, and sulfonylurea are not only linked with weight gain, but also associated with other adverse effects [49]. Weight gain has also been reported as a risk factor for cardiovascular event and increased insulin resistance [50]. Thus, CmNo1 poses an advantage in lowering blood glucose level without affecting body weight.

Insulin resistance is due to an imbalance between hepatic glucose production and tissue uptake [51]. This metabolic disorder follows a decreased glucose uptake in muscle tissue and increased lipolysis resulting in fatty acid accumulation [52], which is coherent with the condition of DM. In addition to improvements in blood glucose uptake (Figure 2), our result showed that CmNo1 treatment also decreased fasting serum insulin levels in HFD-fed mice (Figure 4), indicating insulin utilization by tissues. Fasting insulin measurement has been considered as the most practical and accurate approach for insulin sensitivity [53, 54]. Hence, the results inferred that improvement in blood glucose levels could be due to the positive effect of CmNo1 treatment on insulin receptor sensitivity, providing a new therapeutic avenue for type 2 DM.

To further examine the insulin sensitivity on molecular basis, we analyzed the expression of proteins involved in the insulin-signaling pathway of muscles and adipose tissues. Our results (Figure 5) demonstrated that CmNo1 treatment leads to both the increased phosphorylation and enhanced expression of insulin receptor substrate 1 (IRS-1), protein kinase B (AKT), and glucose transporter type 4 (GLUT-4), indicating that the activated mediators subsequently increased insulin sensitivity. Many previous studies have already demonstrated the activation of IRS-1 and AKT via phosphorylation and GLUT-4 translocation as indicators of insulin sensitivity [55–60]. Additionally, high expression of GLUT-4 has also been reported to promote insulin-mediated glucose uptake [32, 61, 62]. In a nondiseased physiological system, insulin binding leads to receptor conformational change, triggering increased activity of multiple downstream molecules. IRS-1 plays an integral role in the regulation of insulin, acting as a mediator between insulin binding and PI3K/Akt pathway [63]. Similarly, PI3K regulates glucose uptake through the phosphorylation of AKT, a signaling molecule that is required to induce glucose transport [64]. Evidence also supports that malfunction of AKT protein leads to diabetic phenotype [65]. Taken together, activities of these specific mediators have also been demonstrated to measure insulin resistance [66]. Our results pointed out that malfunctioning AKT protein treatment leads to improved blood glucose levels through reverting insulin resistance and allowing glucose to enter cells in muscle and adipose tissue through the insulin signaling pathway.

In addition to the insulin signaling pathway being responsible for hypoglycemic regulation, expression level of peroxisome proliferator-activated receptor *γ* (PPAR-*γ*) was subsequently also analyzed. PPAR-*γ* is a central regulator of insulin sensitization and glucose lowering in adipose tissues and has been suggested as a therapeutic target for DM treatment by drugs such as thiazolidinediones [67, 68]. PPAR-*γ* has also been reported to regulate macrophage-mediated cholesterol efflux and to play a major role in triglyceride lowering [35]. In our study, CmNo1 treatment enhanced PPAR-*γ* protein expression in adipose tissues and led to lowered triglyceride and cholesterol levels (Figure 6). Collectively, based on our results, a mechanistic approach at molecular level has been achieved which corroborates the beneficial effects of* CM* treatment via altering the insulin signaling pathway.

## 5. Conclusions

The CmNo1, a novel combination of fruiting body and mycelia of* CM* for treatment of HFD-induced type 2 DM in a mouse model, led to lowered blood glucose, decreased serum insulin levels, and downregulated expression of PPAR-*γ*. Thus, our result supports the applied combination and its effects on the insulin signaling pathway and major insulin regulator, the PPAR-*γ*. These results indicate that CmNo1 could be a potential and economical therapeutic agent in the treatment of HFD-induced type 2 DM through hypoglycemic activity.

## References

[1] Morales Villegas E. (2006). Syndrome X vs metabolic syndrome. *Archivos de Cardiología de México*.

[2] Tucker K. L., Buranapin S. (2001). Nutrition and aging in developing countries. *The Journal of Nutrition*.

[3] Nakamura T., Terajima T., Ogata T. (2006). Establishment and pathophysiological characterization of type 2 diabetic mouse model produced by streptozotocin and nicotinamide. *Biological and Pharmaceutical Bulletin*.

[4] Szkudelski T. (2012). Streptozotocin-nicotinamide-induced diabetes in the rat. Characteristics of the experimental model. *Experimental Biology and Medicine*.

[5] Boquist L., Hellman B., Lernmark A., Taljedal I. B. (1974). Influence of the mutation ‘diabetes’ on insulin release and islet morphology in mice of different genetic backgrounds. *Journal of Cell Biology*.

[6] Shafrir E. (1996). Development and consequences of insulin resistance: lessons from animals with hyperinsulinaemia. *Diabetes and Metabolism*.

[7] Drel V. R., Mashtalir N., Ilnytska O. (2006). The leptin-deficient (*ob/ob*) mouse: a new animal model of peripheral neuropathy of type 2 diabetes and obesity. *Diabetes*.

[8] Commerford S. R., Bizeau M. E., McRae H., Jampolis A., Thresher J. S., Pagliassotti M. J. (2001). Hyperglycemia compensates for diet-induced insulin resistance in liver and skeletal muscle of rats. *The American Journal of Physiology—Regulatory Integrative and Comparative Physiology*.

[9] Zhang X., Cui Y., Fang L., Li F. (2008). Chronic high-fat diets induce oxide injuries and fibrogenesis of pancreatic cells in rats. *Pancreas*.

[10] Parekh P. I., Petro A. E., Tiller J. M., Feinglos M. N., Surwit R. S. (1998). Reversal of diet-induced obesity and diabetes in C57BL/6J mice. *Metabolism: Clinical and Experimental*.

[11] Hoffler U., Hobbie K., Wilson R. (2009). Diet-induced obesity is associated with hyperleptinemia, hyperinsulinemia, hepatic steatosis, and glomerulopathy in C57Bl/6J mice. *Endocrine*.

[12] Shaul M. E., Bennett G., Strissel K. J., Greenberg A. S., Obin M. S. (2010). Dynamic, M2-like remodeling phenotypes of CD11c+ adipose tissue macrophages during high-fat diet—induced obesity in mice. *Diabetes*.

[13] Reaven G. M. (2005). Why Syndrome X? From Harold Himsworth to the insulin resistance syndrome. *Cell Metabolism*.

[14] Sherwin R. S. (1980). Role of the liver in glucose homeostasis. *Diabetes Care*.

[15] DeFronzo R. A. (1988). Obesity is associated with impaired insulin-mediated potassium uptake. *Metabolism*.

[16] Florez H., Luo J., Castillo-Florez S. (2010). Impact of metformin-induced gastrointestinal symptoms on quality of life and adherence in patients with type 2 diabetes. *Postgraduate Medicine*.

[17] Bell D. S. H., Patil H. R., O'Keefe J. H. (2013). Divergent effects of various diabetes drugs on cardiovascular prognosis. *Reviews in Cardiovascular Medicine*.

[18] Kung J., Henry R. R. (2012). Thiazolidinedione safety. *Expert Opinion on Drug Safety*.

[19] Hollander P. (1992). Safety profile of acarbose, an alpha-glucosidase inhibitor. *Drugs*.

[20] Bando Y., Ushiogi Y., Toya D., Tanaka N., Fujisawa M. (1998). Three diabetic cases of acute dizziness due to initial administration of voglibose. *Internal Medicine*.

[21] Cheung J. K. H., Li J., Cheung A. W. H. (2009). Cordysinocan, a polysaccharide isolated from cultured Cordyceps, activates immune responses in cultured T-lymphocytes and macrophages: signaling cascade and induction of cytokines. *Journal of Ethnopharmacology*.

[22] Ohmori T., Tamura K., Ohgane N. (1989). The correlation between molecular weight and antitumor activity of galactosaminoglycan (CO-N) from Cordyceps ophioglossoides. *Chemical and Pharmaceutical Bulletin*.

[23] Kiho T., Shiose Y., Nagai K., Ukai S. (1992). Polysaccharides in fungi. XXX. Antitumor and immunomodulating activities of two polysaccharides from the fruiting bodies of Armillariella tabescens. *Chemical and Pharmaceutical Bulletin*.

[24] Zhu J.-S., Halpern G. M., Jones K. (1998). The scientific rediscovery of an ancient Chinese herbal medicine: cordyceps sinensis part I. *Journal of Alternative and Complementary Medicine*.

[25] Zhu J.-S., Halpern G. M., Jones K. (1998). The scientific rediscovery of a precious ancient Chinese herbal regimen: cordyceps sinensis: part II. *Journal of Alternative and Complementary Medicine*.

[26] Kiho T., Ookubo K., Usui S., Ukai S., Hirano K. (1999). Structural features and hypoglycemic activity of a polysaccharide (CS-F10) from the cultured mycelium of *Cordyceps sinensis*. *Biological and Pharmaceutical Bulletin*.

[27] Kiho T., Yamane A., Hui J., Usui S., Ukai S. (1996). Polysaccharides in fungi. XXXVI. Hypoglycemic activity of a polysaccharide (CS-F30) from the cultural mycelium of *Cordyceps sinensis* and its effect on glucose metabolism in mouse liver. *Biological and Pharmaceutical Bulletin*.

[28] Kan W. C., Wang H. Y., Chien C. C. (2012). Effects of extract from solid-state fermented *Cordyceps sinensis* on type 2 diabetes mellitus. *Evidence-Based Complementary and Alternative Medicine*.

[29] Hong I., Kang P., Kim K. (2010). Fruit body formation on silkworm by *Cordyceps militaris*. *Mycobiology*.

[30] Hur H. (2008). Chemical ingredients of cordyceps militaris. *Mycobiology*.

[31] Dong Y., Jing T., Meng Q. (2014). Studies on the antidiabetic activities of cordyceps militaris extract in diet-streptozotocin-induced diabetic sprague-dawley rats. *BioMed Research International*.

[32] Wang H.-Y., Kan W.-C., Cheng T.-J., Yu S.-H., Chang L.-H., Chuu J.-J. (2014). Differential anti-diabetic effects and mechanism of action of charantin-rich extract of Taiwanese Momordica charantia between type 1 and type 2 diabetic mice. *Food and Chemical Toxicology*.

[33] Zierath J. R., Kawano Y. (2003). The effect of hyperglycaemia on glucose disposal and insulin signal transduction in skeletal muscle. *Best Practice and Research: Clinical Endocrinology and Metabolism*.

[34] Dimitriadis G., Mitron P., Lambadiari V., Maratou E., Raptis S. A. (2011). Insulin effects in muscle and adipose tissue. *Diabetes Research and Clinical Practice*.

[35] Schoonjans K., Staels B., Auwerx J. (1996). The peroxisome proliferator activated receptors (PPARs) and their effects on lipid metabolism and adipocyte differentiation. *Biochimica et Biophysica Acta—Lipids and Lipid Metabolism*.

[36] Sasase T., Pezzolesi M. G., Yokoi N., Yamada T., Matsumoto K. (2013). Animal models of diabetes and metabolic disease. *Journal of Diabetes Research*.

[37] Hui M. Y., Wang B.-S., Shiow C. H., Duh P.-D. (2006). Comparison of protective effects between cultured Cordyceps militaris and natural Cordyceps sinensis against oxidative damage. *Journal of Agricultural and Food Chemistry*.

[38] Zhang G., Huang Y., Bian Y., Wong J. H., Ng T. B., Wang H. (2006). Hypoglycemic activity of the fungi *Cordyceps militaris*, *Cordyceps sinensis*, *Tricholoma mongolicum*, and *Omphalia lapidescens* in streptozotocin-induced diabetic rats. *Applied Microbiology and Biotechnology*.

[39] Liu X., Huang K., Zhou J. (2014). Composition and antitumor activity of the mycelia and fruiting bodies of *Cordyceps militaris*. *Journal of Food and Nutrition Research*.

[40] Huang S.-J., Tsai S.-Y., Lee Y.-L., Mau J.-L. (2006). Nonvolatile taste components of fruit bodies and mycelia of *Cordyceps militaris*. *LWT—Food Science and Technology*.

[41] Hong I. P., Kang P. D., Kim K. Y. (2010). Fruit body formation on silkworm by *Cordyceps militaris*. *Mycobiology*.

[42] Ahn Y.-J., Park S.-J., Lee S.-G., Shin S.-C., Choi D.-H. (2000). Cordycepin: selective growth inhibitor derived from liquid culture of *Cordyceps militaris* against *Clostridium* spp. *Journal of Agricultural and Food Chemistry*.

[43] Nakamura K., Shinozuka K., Yoshikawa N. (2015). Anticancer and antimetastatic effects of cordycepin, an active component of *Cordyceps sinensis*. *Journal of Pharmacological Sciences*.

[44] Jeong M.-H., Lee C.-M., Lee S.-W. (2013). Cordycepin-enriched *Cordyceps militaris* induces immunomodulation and tumor growth delay in mouse-derived breast cancer. *Oncology Reports*.

[45] Sugar A. M., McCaffrey R. P. (1998). Antifungal activity of 3′-deoxyadenosine (cordycepin). *Antimicrobial Agents and Chemotherapy*.

[46] Kryukov V. Y., Yaroslavtseva O. N., Dubovskiy I. M., Tyurin M. V., Kryukova N. A., Glupov V. V. (2014). Insecticidal and immunosuppressive effect of ascomycete *Cordyceps militaris* on the larvae of the Colorado potato beetle *Leptinotarsa decemlineata*. *Biology Bulletin*.

[47] Shin S., Lee S., Kwon J. (2009). Cordycepin suppresses expression of diabetes regulating genes by inhibition of lipopolysaccharide-induced inflammation in macrophages. *Immune Network*.

[48] Berne R. M. (1980). The role of adenosine in the regulation of coronary blood flow. *Circulation Research*.

[49] Hollander P. (2007). Anti-diabetes and anti-obesity medications: effects on weight in people with diabetes. *Diabetes Spectrum*.

[50] Poirier P., Giles T. D., Bray G. A. (2006). Obesity and cardiovascular disease: pathophysiology, evaluation, and effect of weight loss. *Arteriosclerosis, Thrombosis, and Vascular Biology*.

[51] Saltiel A. R., Kahn C. R. (2001). Insulin signalling and the regulation of glucose and lipid metabolism. *Nature*.

[52] Karpe F., Dickmann J. R., Frayn K. N. (2011). Fatty acids, obesity, and insulin resistance: time for a reevaluation. *Diabetes*.

[53] Panag K. M., Kaur N., Goyal G. (2014). Correlation of insulin resistance by various methods with fasting insulin in obese. *International Journal of Applied and Basic Medical Research*.

[54] Mack R., Skurnick B., Sterling-Jean Y., Pedra-Nobre M., Bigg D. (2003). Fasting insulin levels as a measure of insulin resistance in American blacks. *Journal of Medicine*.

[55] Li H. B., Yang Y. R., Mo Z. J., Ding Y., Jiang W. (2015). Silibinin improves palmitate-induced insulin resistance in C2C12 myotubes by attenuating IRS-1/PI3K/Akt pathway inhibition. *Brazilian Journal of Medical and Biological Research*.

[56] Zhou D., Strakovsky R. S., Zhang X., Pan Y.-X. (2012). The skeletal muscle wnt pathway may modulate insulin resistance and muscle development in a diet-induced obese rat model. *Obesity*.

[57] Avogaro A., de Kreutzenberg S. V., Fadini G. P. (2008). Oxidative stress and vascular disease in diabetes: Is the dichotomization of insulin signaling still valid?. *Free Radical Biology and Medicine*.

[58] Guo S. (2013). Molecular basis of insulin resistance: the role of IRS and Foxo1 in the control of diabetes mellitus and its complications. *Drug Discovery Today: Disease Mechanisms*.

[59] Fuller S., Richard A. J., Ribnicky D. M., Beyl R., Mynatt R., Stephens J. M. (2014). St. John's Wort has metabolically favorable effects on adipocytes in vivo. *Evidence-Based Complementary and Alternative Medicine*.

[60] Tomás E., Lin Y.-S., Dagher Z. (2002). Hyperglycemia and insulin resistance: possible mechanisms. *Annals of the New York Academy of Sciences*.

[61] Leguisamo N. M., Lehnen A. M., Machado U. F. (2012). GLUT4 content decreases along with insulin resistance and high levels of inflammatory markers in rats with metabolic syndrome. *Cardiovascular Diabetology*.

[62] Ikeda S.-I., Tamura Y., Kakehi S. (2013). Exercise-induced enhancement of insulin sensitivity is associated with accumulation of M2-polarized macrophages in mouse skeletal muscle. *Biochemical and Biophysical Research Communications*.

[63] Rung J., Cauchi S., Albrechtsen A. (2009). Genetic variant near IRS1 is associated with type 2 diabetes, insulin resistance and hyperinsulinemia. *Nature Genetics*.

[64] Schultze S. M., Hemmings B. A., Niessen M., Tschopp O. (2012). PI3K/AKT, MAPK and AMPK signalling: protein kinases in glucose homeostasis. *Expert Reviews in Molecular Medicine*.

[65] Garofalo R. S., Orena S. J., Rafidi K. (2003). Severe diabetes, age-dependent loss of adipose tissue, and mild growth deficiency in mice lacking Akt2/PKB*β*. *Journal of Clinical Investigation*.

[66] Leney S. E., Tavaré J. M. (2009). The molecular basis of insulin-stimulated glucose uptake: Signalling, trafficking and potential drug targets. *Journal of Endocrinology*.

[67] Kintscher U., Law R. E. (2005). PPAR*γ*-mediated insulin sensitization: the importance of fat versus muscle. *The American Journal of Physiology: Endocrinology and Metabolism*.

[68] Rocchi S., Auwerx J. (2000). Peroxisome proliferator-activated receptor gamma, the ultimate liaison between fat and transcription. *British Journal of Nutrition*.

