# Corrigendum to “Hypoglycemic Activity through a Novel Combination of Fruiting Body and Mycelia of *Cordyceps militaris* in High-Fat Diet-Induced Type 2 Diabetes Mellitus Mice”

**DOI:** 10.1155/2017/7947401

**Published:** 2017-03-16

**Authors:** Sung-Hsun Yu, Szu-Yu Tina Chen, Wei-Shan Li, Navneet Kumar Dubey, Wei-Hong Chen, Jiunn-Jye Chuu, Sy-Jye Leu, Win-Ping Deng

**Affiliations:** ^1^Graduate Institute of Medical Sciences, College of Medicine, Taipei Medical University, Taipei 110, Taiwan; ^2^Stem Cell Research Center, Taipei Medical University, Taipei 110, Taiwan; ^3^Graduate Institute of Biomedical Materials and Tissue Engineering, College of Oral Medicine, Taipei Medical University, Taipei 110, Taiwan; ^4^Institute of Biotechnology, College of Engineering, Southern Taiwan University of Science and Technology, Yongkang District, Tainan, Taiwan; ^5^Department of Microbiology and Immunology, School of Medicine, Taipei Medical University, Taipei 110, Taiwan

In the article titled “Hypoglycemic Activity through a Novel Combination of Fruiting Body and Mycelia of *Cordyceps militaris* in High-Fat Diet-Induced Type 2 Diabetes Mellitus Mice” [1], there was an error in the first affiliation. The corrected affiliation list is shown above.

## References

[1] Yu S.-H., Chen S.-Y. T., Li W.-S. (2015). hypoglycemic activity through a novel combination of fruiting body and mycelia of *cordyceps militaris* in high-fat diet-induced type 2 diabetes mellitus mice. *Journal of Diabetes Research*.

